# Response Characterization of a Fiber Optic Sensor Array with Dye-Coated Planar Waveguide for Detection of Volatile Organic Compounds

**DOI:** 10.3390/s140711659

**Published:** 2014-07-01

**Authors:** Jae-Sung Lee, Na-Rae Yoon, Byoung-Ho Kang, Sang-Won Lee, Sai-Anand Gopalan, Hyun-Min Jeong, Seung-Ha Lee, Dae-Hyuk Kwon, Shin-Won Kang

**Affiliations:** 1 School of Electronic Engineering, College of IT Engineering, Kyungpook National University, 1370 Sankyuk-dong, Bukgu, 702-701 Daegu, Korea; E-Mails: js1245@ee.knu.ac.kr (J.-S.L.); sw3148@ee.knu.ac.kr (S.-W.L.); gsaianandh@gmail.com (S.-A.G.); hmjeong@ee.knu.ac.kr (H.-M.J.); 2 Department of Sensor and Display Engineering, Kyungpook National University, 1370 Sankyuk-dong, Bukgu, 702-701 Daegu, Korea; E-Mail: duer135@naver.com; 3 Center for Functional Devices Fusion Platform, Kyungpook National University, 1370 Sankyuk-dong, Bukgu, 702-701 Daegu, Korea; E-Mail: bhkang@ee.knu.ac.kr; 4 Department of Biomedical, School of Medicine, Dankook University, 119, Dandae-ro, Dongnam-gu, cheonan-si, 330-714 Chungnam, Korea; E-Mail: shalee@dankook.ac.kr; 5 Department of Electronics Engineering, Kyungil University, Hayang-up, Gyeongsang buk-do 712-702, Korea; E-Mail: dhkwon@kiu.ac.kr

**Keywords:** volatile organic compounds, side-polished optical-fiber, solvatochromic dye, multi-array, gas-sensing, principal component analysis

## Abstract

We have developed a multi-array side-polished optical-fiber gas sensor for the detection of volatile organic compound (VOC) gases. The side-polished optical-fiber coupled with a polymer planar waveguide (PWG) provides high sensitivity to alterations in refractive index. The PWG was fabricated by coating a solvatochromic dye with poly(vinylpyrrolidone). To confirm the effectiveness of the sensor, five different sensing membranes were fabricated by coating the side-polished optical-fiber using the solvatochromic dyes Reinhardt's dye, Nile red, 4-aminophthalimide, 4-amino-N-methylphthalimide, and 4-(dimethylamino)cinnamaldehyde, which have different polarities that cause changes in the effective refractive index of the sensing membrane owing to evanescent field coupling. The fabricated gas detection system was tested with five types of VOC gases, namely acetic acid, benzene, dimethylamine, ethanol, and toluene at concentrations of 1, 2,…,10 ppb. Second-regression and principal component analyses showed that the response properties of the proposed VOC gas sensor were linearly shifted bathochromically, and each gas showed different response characteristics.

## 1. Introduction

Volatile organic compound (VOC) gases are often present in workplaces, especially in chemical industries. It is important to monitor the vapor concentration to safeguard the health of workers by keeping atmospheric emissions in check and preventing environmental hazards [1,2]. With increasing awareness of the hazards to human health, applications related to VOC detection have been attracting much attention in the past few decades.

Several techniques for VOC gas detection have been reported, such as colorimetric gas sensors, semiconductor gas sensors, and optical sensors [3–6]. The colorimetric gas sensor has a simple device structure and allows visual detection, but it cannot precisely detect gas concentrations or allow real-time monitoring [7]. Semiconductor gas sensors based on micro-electromechanical systems (MEMS) and complementary metal oxide semiconductor transistors (CMOSs) can detect precise gas concentrations and allow real-time monitoring, but they need additional heating systems to detach the bound gas molecules, and the sensing membrane must be periodically changed [8,9]. In contrast, optical gas sensors have advantages of high accuracy and sensitivity, fast response time, real-time monitoring, and long-term stability. Thus, optical gas sensors are widely used in the field of environmental science [2,10,11].

Recently, side-polished optical-fiber sensors have been shown to be good candidates for monitoring environmental changes because they not only have mechanical stability, but also sensitivity control by changing the planar waveguide materials [12–17].

In our previous work, we confirmed that side-polished optical-fiber sensors can be used to detect the VOC gas xylene using a solvatochromic dye [14]. Further, we adopted a multi-sensor array based on the pulse-width modulation principle to detect various VOC gases. The pulse-width of the signal received from the optical-fiber waveguide depends on the absorption of the evanescent field by the gas on the polished cladding region [16,17]. The proposed sensing system has a wide dynamic range but is a very complex system with long response and recovery times.

Here, we propose a simple multi-array VOC gas detection system based on changing the effective refractive index. The different polarities of the VOC gases cause changes in the effective refractive index of the sensing membrane owing to evanescent field coupling. We fabricated sensing membranes by incorporating five different solvatochromic dyes into poly(vinylpyrrolidone) (PVP) polymer. Upon contact between the VOC gases and the sensing membrane, the refractive index changes on the basis of the charge-transfer (CT) characteristics of the solvatochromic dye in the device's polymer planar waveguide (PWG). Our proposed optical-fiber gas sensor has a good linear response over a broad dynamic range and can detect approximately 10 ppb of VOC gas. Principal component analysis (PCA) was used to explore the data distribution and classify the VOCs [18,19].

## 2. Experiment Materials and Methods

### 2.1. Fabrication of Side-Polished Optical-Fiber Device

**Figure 1.**
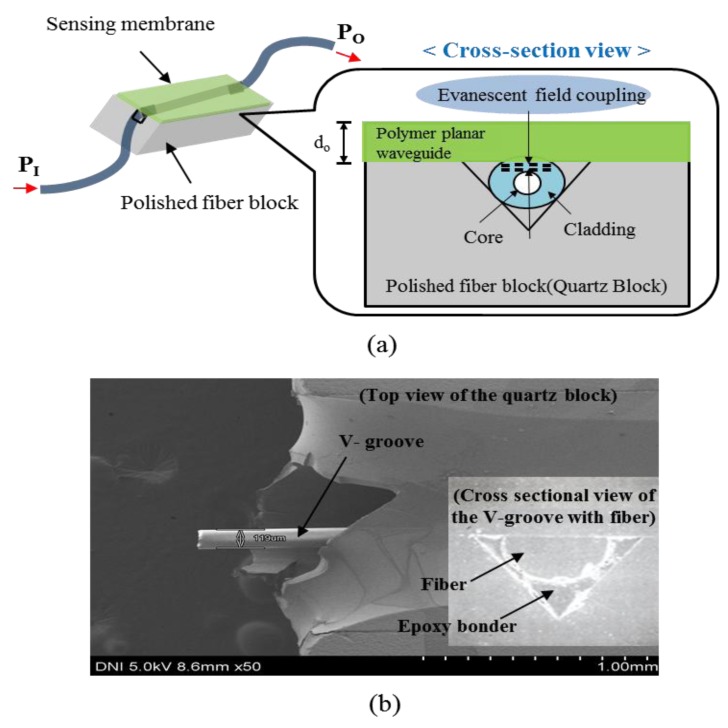
Side-polished optical-fiber device: (**a**) schematic diagram; and (**b**) SEM image.

The side-polished optical-fiber device was fabricated by fiber polishing a single-mode optical-fiber fixed in a 160 μm wide V-groove in quartz block (5 × 10 × 25 mm^3^). The V-groove was fabricated using a mechanical slicer. A single-mode optical-fiber of core radius 3 μm and cladding radius 125 μm was placed in the V-groove. The bending radius of the optical-fiber inside the quartz block was approximately 50 cm. The final stage of the sensing device fabrication process was fiber polishing [20]. A schematic diagram with a scanning electron microscope (SEM; S-4800, Hitachi. Ltd, Ibaraki, Japan) image of the proposed side-polished optical-fiber is shown in Figure 1.

### 2.2. Solvatochromism and Solvatochromic Dyes

**Figure 2.**
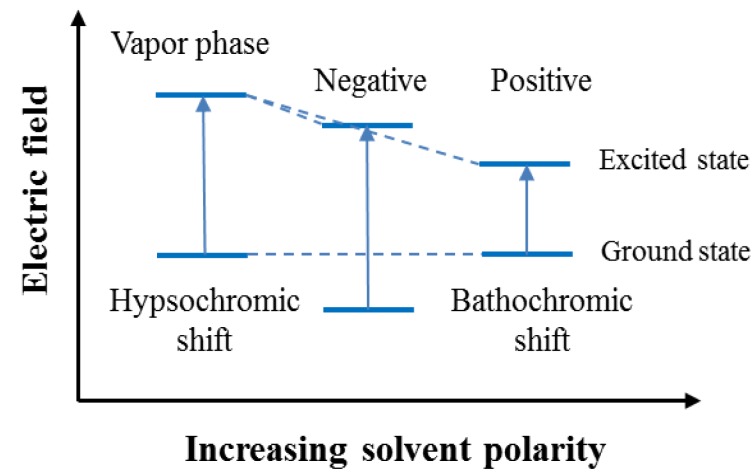
Schematic diagram of solvatochromism.

Solvatochromism is the phenomenon whereby a chemical substance changes color due to changes in the solvent polarity of the surrounding environment. A bathochromic (red) shift of the ultraviolet/visible absorption band with increasing solvent polarity is called “positive solvatochromism.” The corresponding hypsochromic (blue) shift [21,22] with increasing solvent polarity is called “negative solvatochromism.” A schematic diagram of solvatochromism is shown in Figure 2.

Dyes that exhibit solvatochromism are called solvatochromic dyes or solvatochromic compounds. A solvatochromic effect or solvatochromic shift refers to a strong dependence of the absorption and emission spectra of a compound on the solvent polarity [22]. Because the polarities of the ground and excited states of a chromophore are different, a change in the solvent polarity leads to differential stabilization of the ground and excited states, and therefore a change in the energy gap between these electronic states. Consequently, variations in the position, intensity, and shape of the absorption spectrum can be direct measures of the specific interactions between the solute and solvent molecules.

### 2.3. Fabrication of Sensing Membrane

**Figure 3.**
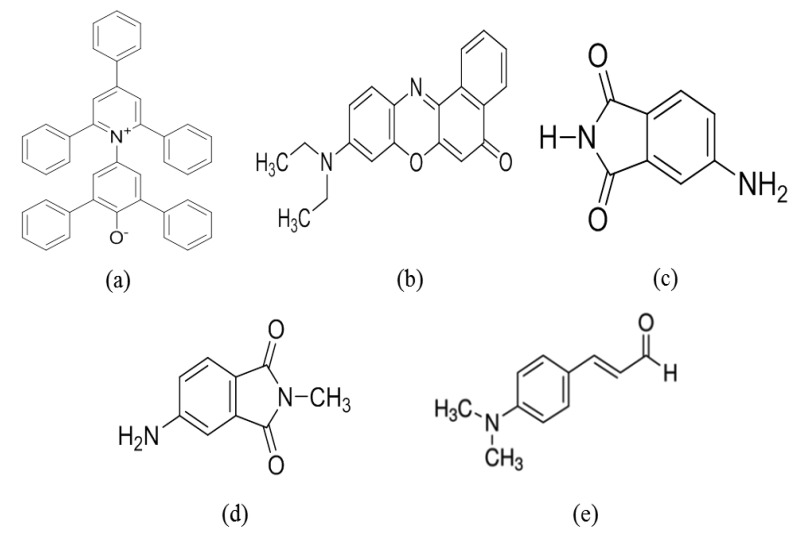
Molecular structure of solvatochromic dye: (**a**) Reichardt's dye; (**b**) Nile red dye; (**c**) AP dye; (**d**) 4-ANMP dye; and (**e**) DMACA dye.

**Figure 4.**
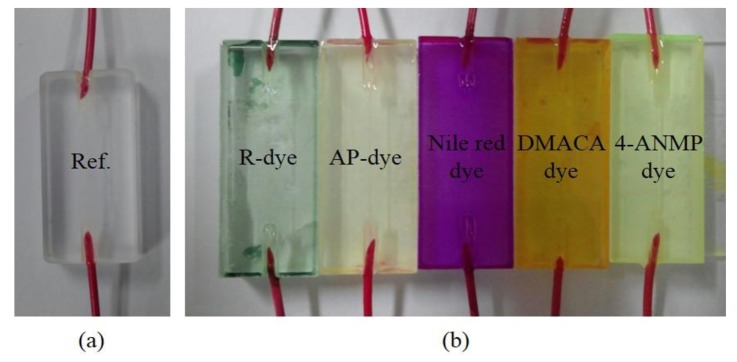
Photograph of the fabricated side-polished optical-fiber device (**a**) before and (**b**) after depositing the sensing membrane.

A solvatochromic dye was used on the sensing membrane [22]. The refractive index of the sensing membrane changed depending on the CT characteristics. A sensor array consisting of five different solvatochromic dyes, such as Nile red [21], Reichardt's dye (R-dye) [22], 4-aminophthalimide (AP) [23,24], 4-amino-N-methylphthalimide (4-ANMP) [25], and 4-(dimethylamino)cinnamaldehyde (DMACA) [26] was tested. The molecular structures of the solvatochromic dyes are shown in Figure 3. The sensing membrane was prepared by mixing 2 wt% solvatochromic dye with 38 wt% PVP [27] dissolved in N,N-dimethylacetamide (99%) [28]. All reagents were purchased from the Sigma-Aldrich. Co. LLC. (St. Louis, MI, USA). The mixture was sonicated for 10 min to obtain the sensing solution. The sensing membrane was coated on the side-polished optical-fiber block, by using the optimized spin-coating thickness conditions. After fabrication of the sensing membrane, the fiber block was dried overnight at room temperature. The thickness of the sensing membrane was determined using SEM, and was approximately 2.2 μm. The fabricated side-polished optical-fiber VOC-sensing elements before and after spin-coating of the sensing membrane are shown in Figure 4.

### 2.4. VOC Gas Detection System

**Figure 5.**
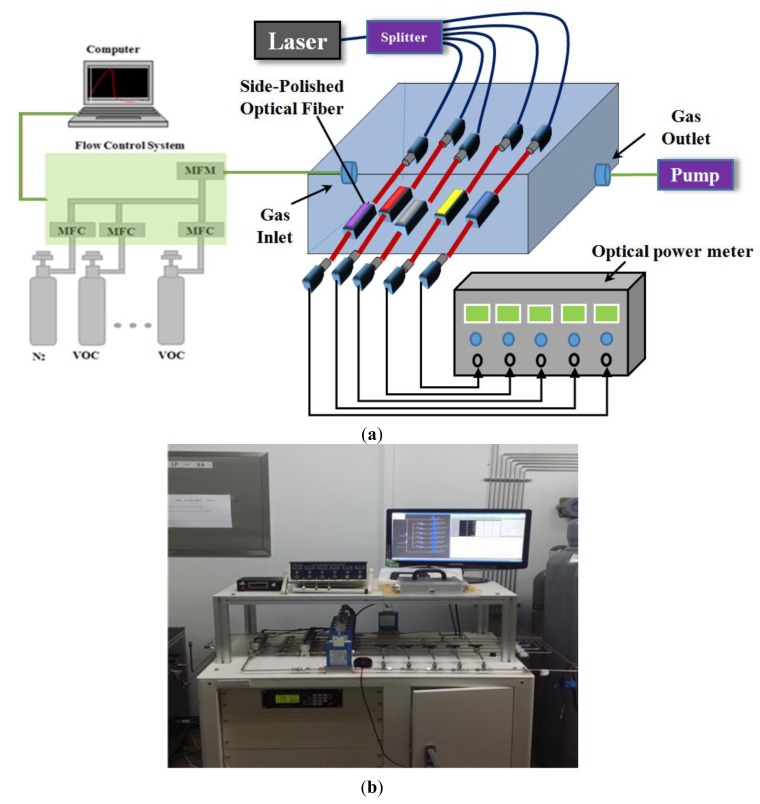
(**a**) The schematic diagram and (**b**) photograph of the multi-array VOCs gas detection system.

The layout of the VOC gas detection system is depicted in Figure 5. The experimental setup is based on a change in the refractive index, similar to previously reported setup applied to the characterization of wavelength modulation [14]. The VOC detection system consisted of a multi-array gas chamber (50 × 150 × 250 mm^3^), five side-polished optical-fiber VOC sensing elements, a gas cabinet (VOCs and nitrogen), power supply, and fiber coupled laser module consisting of a combination of a single-mode laser diode controller (850 nm), splitter (FCLT-2416-11B251), optical power meter (zAB003), mass flow controllers (MFCs), mass flow meter (MFM), and a computer. The optical input of the laser diode controller was passed through the splitter to each of the devices. The optical output in the fabricated gas chamber from the devices simultaneously exposed to VOC gases was measured using the power meter.

The VOC gases were emitted from the gas cabinet, so the precise concentration of the gases could be controlled using computerized MFCs and MFM. MFCs are used to measure and control the flow of fluids and gases, and are designed, calibrated to control a specific type of fluid or gas over a particular range of flow rates.

The MFC can be set at 0% to 100% of its full-scale range, but is typically operated in the 10% to 90% range, where the best accuracy is achieved. In the absence of VOC gas in the gas chamber, the system was adjusted so that almost equal amounts of light passed through the sensing optical-fiber, and there was no change in the refractive index. On the other hand, upon exposure to VOC gases, the refractive index of the PWG changed. The gases acetic acid, benzene, dimethylamine, ethanol, and toluene were detected at concentrations that were varied from 1 to 10 ppb in 1 ppb steps, and the detection limit was 1 ppb which is the detection system limit.

The VOC gases were mixed with nitrogen gas (99.999%) to give the desired concentration. N_2_ is an inert gas and does not react with VOCs or the components of the sensing membrane [29]. All the gas-detection experiments were performed under ambient conditions. The VOC gas-detection system was connected to the computer. The processing unit was used to monitor real-time response to record the results.

## 3. Results and Discussion

**Figure 6.**
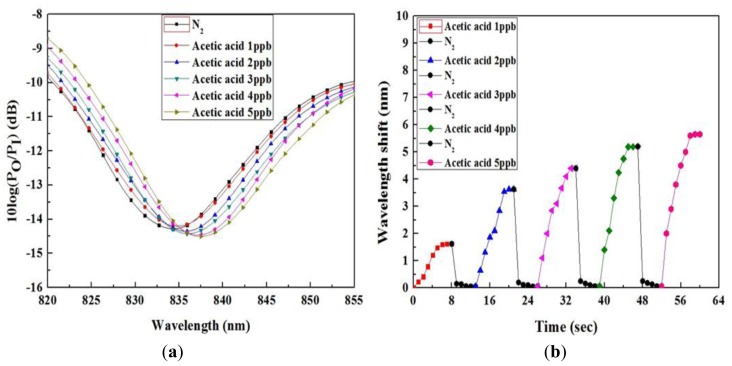
Spectrum analyzes results of after acetic acid gas exposure: (**a**) variations in resonance wavelength shift; and (**b**) saturation and recovery characteristics.

To observe the saturation and recovery time of the AP-dye coated sensing membrane of the sensor array for acetic acid gas, we injected acetic acid gas at concentrations varying from 1 to 5 ppb into the gas chamber. The saturation and the recovery times of the proposed VOC gas-detection system were 8 and 5 s, respectively, at room temperature. Figure 6 shows the variation in resonance wavelength shift after acetic acid gas exposure, and the saturation and recovery characteristics of the sensor. We compared the performance of the proposed detection system with that of similar detection systems [14,16,30–32]. Among these methods, the response and recovery times reported in [16] were less than 30 s, whereas in our proposed detection system, the response and recovery times were less than 10 s. The comparison of analytical performance of similar detection systems is given in Table 1.

In order to prepare and calibrate the sensor, N_2_ inert gas was allowed to flow into the gas chamber of the detection system. Then, the resonance wavelength of side-polished optical-fiber was fixed at a wavelength of 850 nm because of the stable baseline.

**Figure 7.**
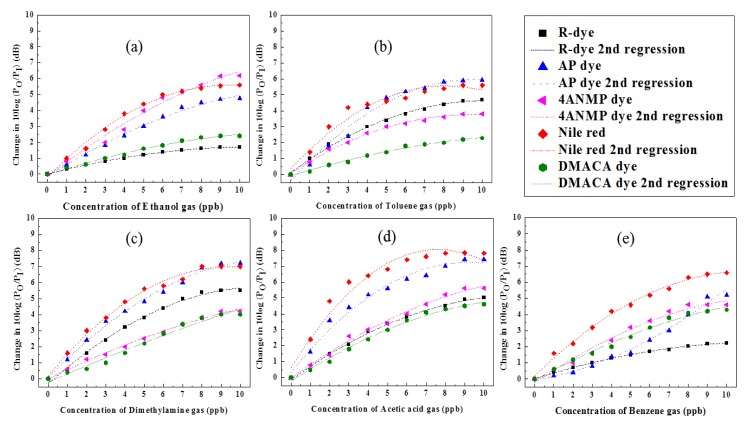
The resonance spectrum shift and second-regression of sensing membrane according to volatile organic compound gas concentration: (**a**) ethanol; (**b**) toluene; (**c**) dimethyalmine; (**d**) acetic acid; and (**e**) benzene.

In our system in resonance wavelength changes when the refractive index of the PWG is changed. In our system when we flow 0.5 ppb of VOC gas into the gas chamber then the refractive index of the PWG shows a negligible change, which corresponds to the negligible shift of the resonance wavelength. But, when we flow 1 ppb of VOC gas into the gas chamber then the resonance wavelength changed considerably. That's why we consider the detection of limit the system is 1 ppb. The VOC gas was injected systemically and concentrations varied from 1 to 10 ppb in the interval of 1 ppb to verify the sensitivity and resolution. To determine the selectivity of the sensor, the sensor array distinguishes the different VOC gases on the basis of the responses of the five different sensing membranes. The wavelength shift of each sensing membrane for the five different VOC gases are shown in Figure 7.

The results indicate that the relative resonance wavelength shifts as the concentration of the VOCs gases increases. The dynamic range was approximately from 1 to 8 ppb and saturation level for VOC gas detection was ∼9 ppb. We repeated the experimental measurements about three times under VOC gas concentrations ranging from 1 to 10 ppb. Thus, we confirmed the performance of the proposed detection system by repeating similar experiments several times. As a result, the R-squared value [R^2^] of the wavelength shift exhibited a good dynamic range from 0.95344 to 0.99885 [33].

**Figure 8.**
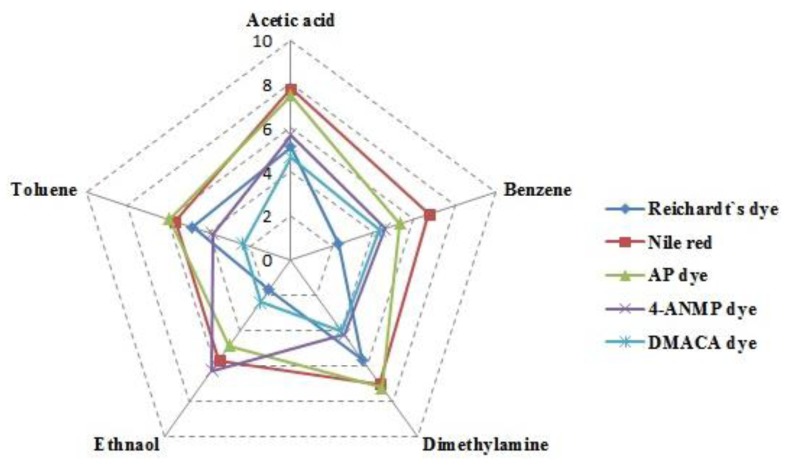
Average response polar plot of various VOC gases.

The average gas-sensing responses of the five different VOC gases for different sensing membranes are shown in Figure 8, using a radar chart. The ethanol sensitivity of the membrane coated with 4-ANMP dye is higher than its sensitivities for other VOC gases, whereas the AP-dye coated sensor has a high sensitivity for toluene and dimethylamine gases, and the Nile red dye coated sensor has high sensitivity for acetic acid and benzene gases. In the case of Nile red sensing membrane, when we flowed same concentrations of acetic acid and ethanol gas individually into the gas chamber the acetic acid gas showed higher sensitive than the ethanol gas because acetic acid is more polar than the ethanol. As a result, in the case of acetic acid the refractive index of the Nile red sensing membrane changes more than in the case of ethanol gas which causes a bigger shift of the resonance wavelength than in the case of ethanol gas. The sensitivities of the R-dye containing sensor are lower than those of the other sensing elements.

**Figure 9.**
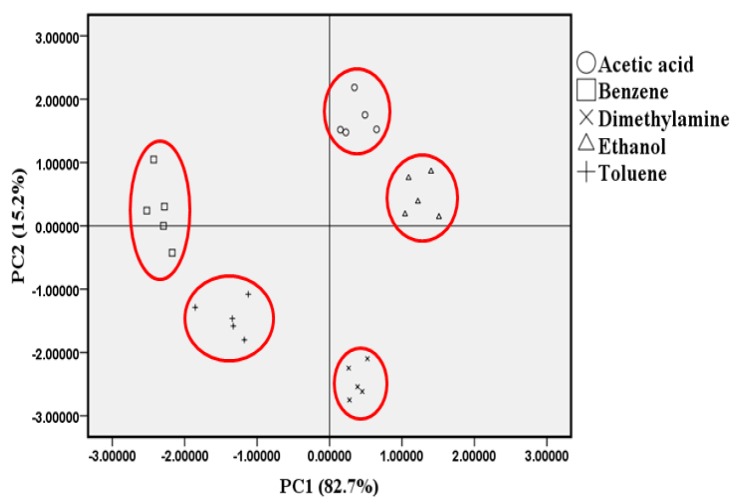
PCA plot of various VOC gases detected by side-polished optical-fiber.

We used PCA to determine the pattern of discrimination and separation of the proposed VOC gas-detection system. PCA is a Statistical Package for Social Science (SPSS) tool used to analyze, classify, and reduce the dimensionalities of numerical datasets in multivariate problems [34]. The first component extracted in PCA, PC1, includes the maximum total variance among the observed variables. This means that PC1 is correlated with at least some of the observed variables. The second component, PC2, includes the variance in the data set that was not accounted for by the first component [35]. The second component is not correlated with the first component. Figure 9 shows the two-dimensional PCA (PC1–PC2) plot of the sensing discrimination for various VOC gases. PC1 explains 82.7% of the variance and PC2 explains 15.2% of the variance, and together they explain 97.9%. The sensor array distinguishes the different VOC gases on the basis of the responses of the five different sensing membranes. The separate ellipses, including the measurements for single VOC gases, indicate that the five primary VOCs (acetic acid, benzene, dimethylamine, ethanol, and toluene gases) can be clearly discriminated from each other. These results indicate that the proposed side-polished optical-fiber VOC gas sensor array can successfully separate different VOC gases, even at low concentrations.

## 4. Conclusions

We have developed a new design for a highly sensitive optical gas sensor to detect VOC gases using evanescent-field coupling between a side-polished optical-fiber and a PWG. Five different solvatochromic dyes (R-dye, Nile red, AP, 4-ANMP, and DMACA) and a PVP polymer were used to fabricate five different types of sensing membranes. The proposed VOC gas sensor response characteristics were reproducible, and the linear response of the detection system was good. The types and quantities of VOC gases were determined in real-time using the proposed detector. The response and recovery times were less than 10 s. PCA was used to distinguish different VOC gases on the basis of the responses of the five different sensing membranes. The proposed VOC gas sensor has many advantages, including easy fabrication, good selectivity, short response time, linear response over a practical range, low cost, and high sensitivity. We believe that sensor devices produced using the same fabrication process under the same conditions would show good stability. In the future, we plan to extend the analysis to include other VOC gases in mixed-gas experiments using different sensor array systems.

## Figures and Tables

**Table 1.** Comparison of the analytical performance of similar VOC detection system.

**Principle of Measurement**	**Dynamic Range (ppm)**	**Detection Limit (ppm)**	**Resolution (ppm)**	**Response Time (s)**	**Recovery Time (s)**
Refractive index change	0∼0.01	0.001	0.001	10	5
Refractive index change [14]	0.02∼1	0.02	0.010∼0.2	60	60
Pulse-width modulation [16]	0∼0.05	0.01	0.01	25	30
Relative reflectance [30]	1∼10	1	1∼2	1800	3600
Adsorption/catalytic combustion [31]	0.01∼1	0.01	0.01∼0.3	1200	1200
Relative voltage [32]	15∼1000	15	15∼500	700∼800	<100
